# Death receptor-based enrichment of Cas9-expressing cells

**DOI:** 10.1186/s12896-016-0250-4

**Published:** 2016-02-16

**Authors:** C. Liesche, L. Venkatraman, S. Aschenbrenner, S. Grosse, D. Grimm, R. Eils, J. Beaudouin

**Affiliations:** Department for Bioinformatics and Functional Genomics at the Institute of Pharmacy and Molecular Biotechnology and BioQuant BQ0020, University of Heidelberg, and Division of Theoretical Bioinformatics, German Cancer Research Center (DKFZ), Im Neuenheimer Feld 267, 69120 Heidelberg, Germany; Department of Infectious Diseases/Virology, Cluster of Excellence CellNetworks, Heidelberg University Hospital, BioQuant BQ0030, Im Neuenheimer Feld 267, 69120 Heidelberg, Germany

**Keywords:** CRISPR/Cas9, Genome editing, T7E1, Indel, Editing efficiency

## Abstract

**Background:**

The CRISPR/Cas9 genome editing system has greatly facilitated and expanded our capacity to engineer mammalian genomes, including targeted gene knock-outs. However, the phenotyping of the knock-out effect requires a high DNA editing efficiency.

**Results:**

Here, we report a user-friendly strategy based on the extrinsic apoptosis pathway that allows enrichment of a polyclonal gene-edited cell population, by selecting Cas9-transfected cells that co-express dominant-negative mutants of death receptors. The extrinsic apoptosis pathway can be triggered in many mammalian cell types, and ligands are easy to produce, do not require purification and kill much faster than the state-of-the-art selection drug puromycin. Stringent assessment of our advanced selection strategy via Sanger sequencing, T7 endonuclease I (T7E1) assay and direct phenotyping confirmed a strong and rapid enrichment of Cas9-expressing cell populations, in some cases reaching up to 100 % within one hour. Notably, the efficiency of target DNA cleavage in these enriched cells reached high levels that exceeded the reliable range of the T7E1 assay, a conclusion that can be generalized for editing efficiencies above 30 %. Moreover, our data emphasize that the insertion and deletion pattern induced by a specific gRNA is reproducible across different cell lines.

**Conclusions:**

The workflow and the findings reported here should streamline a wide array of future low- or high-throughput gene knock-out screens, and should largely improve data interpretation from CRISPR experiments.

**Electronic supplementary material:**

The online version of this article (doi:10.1186/s12896-016-0250-4) contains supplementary material, which is available to authorized users.

## Background

The CRISPR/Cas9-system has become an extremely powerful tool for the editing of genes in various cell types. The method was born thanks to the study of clustered, regularly interspaced, short palindromic repeat (CRISPR)-genes and CRISPR-associated (Cas) genes of bacteria, which constitute part of the bacterial defense mechanism [1, 2]. As a tool in molecular and cell biology, the CRISPR/Cas9-system is employed by co-expressing a single guide RNA (gRNA), which targets a DNA sequence of interest, and the Cas9 nuclease, which can bind to the gRNA and produce double-strand breaks [3–6]. Repeated cycles of cut and faulty repair by the cellular non-homologous end joining machinery can eventually generate insertion/deletion (indel) mutations, and consequently a knock-out effect in cases where these mutations introduce a frame-shift within the targeted gene.

By targeting DNA, the CRISPR/Cas9 system perfectly complements gene knock-down on the RNA level by RNA interference for the annotation of gene functions [7]. Importantly, as compared to siRNA/shRNA technology, phenotyping by gene knock-out is not constrained by a reduction of gene expression that can be incomplete and that can vary from cell to cell. From this perspective, the technique will be particularly useful to screen for gene functions. CRISPR/Cas9-based screens have so far used pooled libraries, in situations where the identification of hits was allowed by the isolation of cells showing the right phenotype [8–12]. Arrayed screens can offer a broader range of phenotyping possibilities [13] which can go up to subtle changes at the cellular scale that can only be observed by microscopy. Therefore, combining the pipelines existing for arrayed RNAi screens [14] and the CRISPR/Cas9technology should provide a highly sensitive tool to determine gene functions. Still, a technical limitation of the CRISPR/Cas9-system is the efficiency of gene knock-out within a cell population, which remains a limiting and highly variable factor. For example, the first studies validating the CRISPR approach for mammalian cells demonstrated proportions of gene-edited cellular subpopulations ranging between 2 and 25 %, depending on the cell type and the mode of Cas9/gRNA transfection [3, 6, 15]. Two main strategies have improved the efficiency, the first being the optimization of the delivery method, using e.g. lentiviral, adenoviral or Adeno-associated viral (AAV) vectors [16]. Lentivirus libraries have successfully been used for various screening projects [8–12] and AAVs have been applied in animals [17–19]. Potential drawbacks of this viral vector strategy are the requirement to work under elevated biosafety levels (lenti- and adenoviruses), the stable chromosomal integration of the viral genomes encoding the Cas9 nuclease (lentiviruses), or the limited capacity for packaging of foreign DNA such as Cas9 cDNAs (AAV). The other strategy consists of selecting, enriching and expanding cells from a cell population by using co-expressing markers [20], as briefly summarized in the following. Co-expression of fluorescent proteins [21, 22] or of reporter plasmids exhibiting fluorescent protein expression after editing of the reporter gene [23, 24] allows for easy and robust selection by flow cytometry sorting. Nevertheless, its application in arrayed screens would be laborious. Another selection strategy that is more amenable for a screening strategy consists of killing cells that are not expressing Cas9. This was performed either by co-expressing Cas9 and a gene that provides resistance to a drug [20, 25, 26] or by co-editing a gene of interest and a gene that would induce cell death upon treatment with a drug. This last approach was performed by targeting the HPRT gene and by using 6-TG as death inducer [27]. The fluorescence- and drug-based approaches have the advantage of relying on transient expression of Cas9, and therefore can be considered as scarless: the only genome modification that is left after the transient expression is the editing event itself, but no Cas9/gRNA insertion or acquired drug resistance. Regarding the selection by cell killing, the process should occur within two days, during the typical peak of expression of the resistance gene. For this reason, puromycin is typically preferred to other drugs as it generally kills cells within 24 to 48 h. Provided a good balance between killing of non-transfected cells and survival of transfected cells, one can yield efficient genome editing even in the case of suboptimal transfection with limited cost and material.

Here, we aimed at enlarging the panel of tools for potent and fast enrichment of gene-edited cells, and thus implemented an original strategy based on killing of transfected cells that lack Cas9 expression through the extrinsic apoptosis signaling pathway. The activation of the TNF receptor family members FAS/CD95 by CD95 ligand (CD95L), or DR4 and DR5 by TRAIL, can induce cell death in many cellular contexts through the activation of caspases [28–30]. This activation occurs through the recruitment of the adaptor protein FADD on the death domain (DD) of oligomerized, ligand-associated receptors [31]. Because of this oligomerization step, receptor mutants that lack the DD can block apoptosis even in the presence of wild type (wt) receptors, in a dominant-negative manner [32]. We therefore hypothesized that co-expression of the Cas9 nuclease with such receptor mutants would render cells resistant to CD95L- or TRAIL-induced apoptosis, and that incubation with the appropriate ligand would allow for the enrichment of Cas9-positive cells with no further modification of the cells. In vitro, killing cells can be efficiently achieved by using modified forms of soluble CD95 and TRAIL ligands that are, for example, fused to an isoleucine zipper [33]. Ligands can be produced in-house in a straightforward and inexpensive manner, do not require purification and kill cells within a few hours.

For proof-of-concept, we successfully applied our method to the p65 subunit of NFκB, IRF3 and TLR3, using three different gRNAs per gene. By using the T7E1 assay and Sanger sequencing, we obtained estimations of up to 100 % gene-edited cells. As a result of this enrichment, we could directly show the involvement of the three genes in Poly (I:C)-induced cell death in HeLa cells. Moreover, we observed that a given gRNA generated a reproducible indel pattern in different cell lines. This pattern showed a limited complexity, which had a strong influence on the calculation of editing efficiency from the T7E1 assay. We conclude that our strategy extends the panel of tools for the CRISPR/Cas9 technology and can be particularly applicable to screens as it is easy to implement and reduces the time for the selection of gene-edited cells.

## Results

### Death receptor-based selection of Cas9-expressing cells

**Fig. 1:**
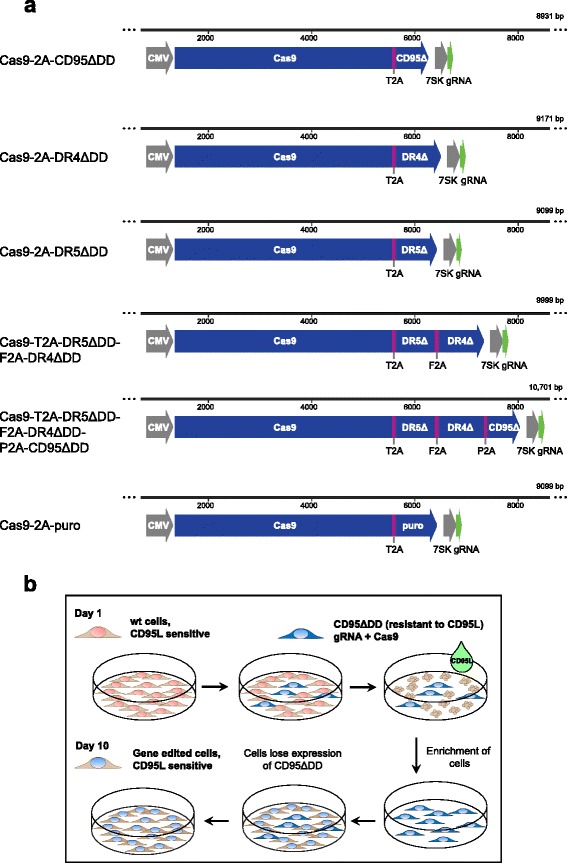
Constructs and workflow of death receptor-based enrichment of Cas9-expressing cells. **a** Constructs for different selection strategies. The Cas9 nuclease is linked to the resistance gene by a cleavable 2A peptide allowing stoichiometric expression. As resistance gene, we used death receptors that lack the intracellular death domain, denoted by ∆. CD95 is chosen when using CD95L as selection agent. DR4 and DR5 are death receptors that bind the death ligand TRAIL. To allow simultaneous expression of several death receptors, we linked them to Cas9 via the 2A peptide from *Thosea asigna* virus (T2A), from *foot-and-mouth disease virus* (F2A) and *porcine teschovirus-1* (P2A). To allow comparison to the puromycin selection strategy, we cloned the puromycin resistance gene puromycin *N*-acetyl-transferase, denoted here as puro. We used the human cytomegalovirus (CMV) and the 7SK promoters for the protein and gRNA expression, respectively. **b** Workflow. Cells are transfected with a plasmid encoding the Cas9 nuclease, a selection gene and the gRNA. Two days after transfection, death ligand was added to the cells, before they were washed and expanded

We established a death receptor-based selection system that enriches Cas9-expressing cells in order to generate polyclonal, gene-edited cell populations. To this end, we designed a single plasmid encoding gRNA and Cas9 nuclease together with the selection gene that is co-expressed via a 2A peptide, adapted from [10] (Fig. 1a). Using death ligands as selection agent, we aimed at killing non-transfected cells and cells expressing too low levels of Cas9 nuclease. As selection genes, we used truncations of death receptors that are unable to transmit the apoptotic signal (see workflow on Fig. 1b). More precisely, we made truncated receptors missing their death domain (ΔDD) for the death ligand CD95 ligand (CD95-ΔDD) or TRAIL (DR4-ΔDD and DR5-ΔDD) (Fig. 1a). We tested their single expression as well as the simultaneous expression of both, DR4-ΔDD and DR5-ΔDD, or even of all three mutant receptors, CD95-ΔDD, DR4-ΔDD and DR5-ΔDD, from the same construct. As detected by immunofluorescence, all our vectors robustly over-expressed the mutant receptors in the different cell lines that were tested, HeLa, HT-1080, LN-18 and MDA-MB-231 (Additional file 1: Figure S1-S4).

**Fig. 2:**
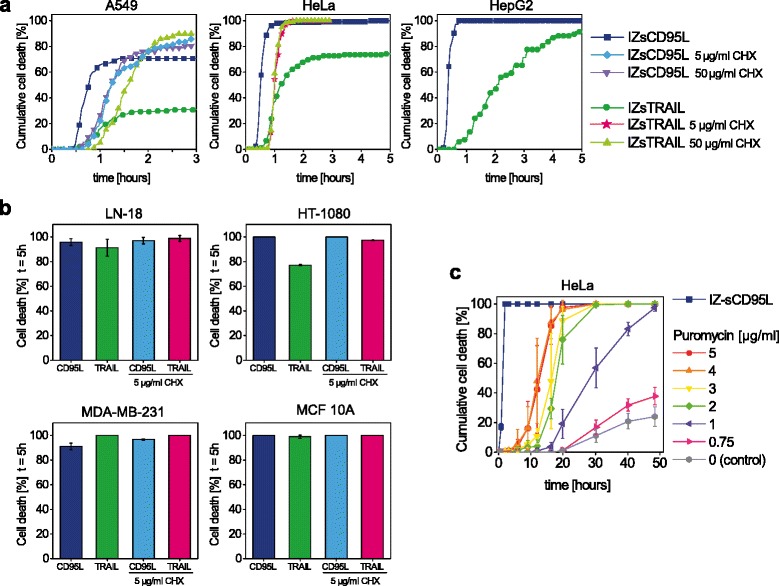
Death ligands induce cell death in various cell types within a few hours. Cell death was measured by observing cell morphology by microscopy. **a** Cell death kinetics of A549, HepG2 and HeLa cells using IZsTRAIL and IZsCD95L, with and without co-treatment with 5 to 50 μg/ml cycloheximide (CHX). **b** Cell death of LN-18, HT-1080, MDA-MB-231 and MCF10A cells treated for 5 h with IZsCD95L (denoted CD95L) or IZsTRAIL (denoted TRAIL), with or without 5 μg/ml CHX. **c** HeLa cell death kinetics comparison between IZsCD95L and puromycin. While cell death occurred after 2 h with IZsCD95L, it saturated at 30 h with puromycin. b and c: mean ± s.d. of 3 different fields of view with at least 50 cells each

As we aimed at developing a strategy that would minimize the requirements for production of reagents, we next tested the possibility to directly use secreted, non-purified ligands expressed in HEK 293 T cells. We measured the apoptotic activity of soluble death ligands (sCD95L and sTRAIL) that are fused to the isoleucine-zippper domain (IZ), denoted as IZsCD95L and IZsTRAIL, in different human cell lines (including those from Additional file 1: Figure S1-S4): HeLa, HT-1080, LN-18, MDA-MB-231, HepG2, MCF10A and A549 (Fig. 2a and b). With the exception of A549, all cell lines showed more than 99 % cell death within 5 h with at least one of the ligands. Remarkably, 1 h was even sufficient to kill HeLa and HepG2 cells. Addition of 5 or 50 μg/μl cycloheximide, known to amplify the sensitivity to death receptors [34], could further enhance cell death in some cases. Still, in all cells except A549, one of the two ligands efficiently induced cell death without cycloheximide (Fig. 2), showing that those ligands can be directly used as strong selection agent. Notably, cell death induced by puromycin, even at saturating concentrations, required about 30 hours to kill more than 99 % HeLa, LN-18 and MDA-MB-231 cells (Fig. 2c and S5). Therefore, cell death can be more efficiently induced by death ligands than by puromycin.

### Estimating genome editing efficiency in HeLa cells by Sanger sequencing and T7E1 assay

**Fig. 3:**
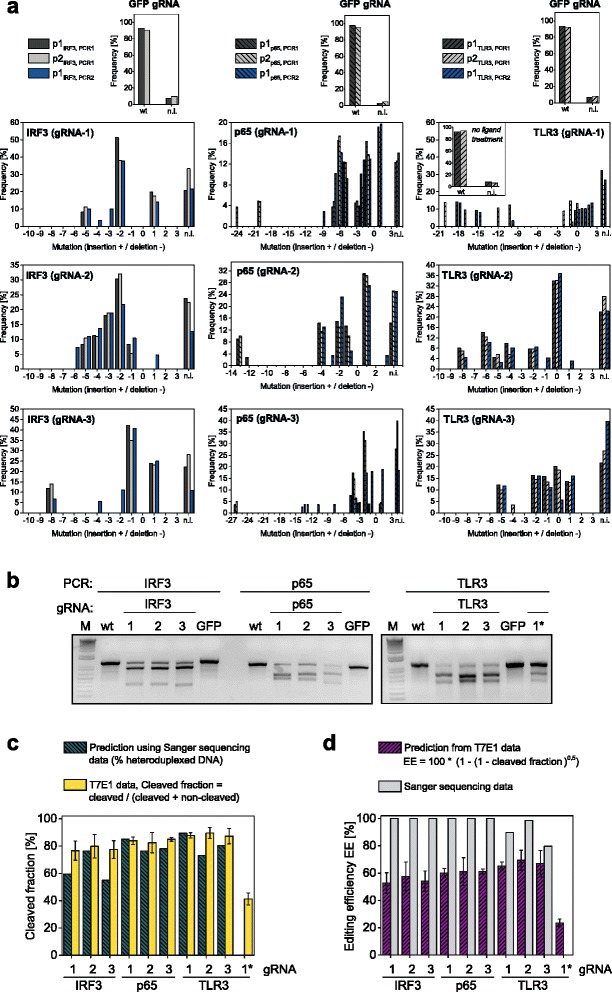
Editing of IRF3, p65 and TLR3 genes in HeLa cells using the Cas9-T2A-CD95∆ construct and IZsCD95L as selection agent. Three different gRNAs per gene were tested and a control gRNA targeting GFP was used. Cells denoted as *TLR3* gRNA-1* were not treated with IZsCD95L. **a** Sanger sequencing results. The frequency of indels in polyclonal cell lines was quantified from chromatograms using the TIDE analysis. Genome extraction, PCR and sequencing were performed twice. PCR1 was in addition sequenced with a second primer (p2). Mutatio*n* = 0 represents wild type sequence. The non-interpretable fraction (n.i.) relates to the correlation coefficient of the TIDE analysis with R^2^ = 100 - n.i. **b** Representative agarose gels from three T7E1 assays. Genome extraction from polyclonal HeLa cell lines, PCR and T7E1 digest were repeated three times. **c** Predictions of the cleavage fraction were obtained by estimating the amount of heteroduplexed DNA by using equation (2) and data from the Sanger sequencing (green-striped). The cleavage fraction (yellow bars) was quantified from T7E1 assays by: cleaved DNA/(cleaved DNA + non-cleaved DNA). **d** Purple-striped bars show the predicted editing efficiency using equation (1) and data from the T7E1 assay. A different estimate of the editing efficiency was obtained by Sanger sequencing and the TIDE analysis (grey bars)

In order to test the capacity of our death receptor strategy to efficiently generate gene-edited cells, we targeted three different genes in HeLa cells, *IRF3*, *TLR3* and *p65*, with three different gRNAs for each gene. As CD95L killed HeLa cells more efficiently than TRAIL (Fig. 2a), we used the Cas9-2a-CD95ΔDD construct. As mock control, we used a gRNA that was designed to target *gfp* (see Table 1 for the primer and gRNA sequences used in this study). Two days after transfection, cells were incubated for 5 h with IZsCD95L. We also kept cells transfected with *TLR3* gRNA-1 untreated, denoted as *TLR3* gRNA-1*. Seven days after treatment, genomic DNA was extracted for analysis and the remaining cells were kept in culture for direct phenotyping. We evaluated the editing efficiency, denoting the fraction of mutant DNA species, by using two different methods, namely the analysis of Sanger sequence chromatograms (Fig. 3a) and the T7E1 assay (Fig. 3b). To quantify the mutations from sequencing chromatograms, we applied the TIDE (Tracking of Indels by DEcomposition) analysis, a sequence decomposition approach [35]. To this end, we PCR-amplified the genomic region targeted by the different gRNAs in the polyclonal HeLa cell lines. The three gRNAs for one gene were located in the same region of the genome, hence we used the same primers for each gene. To check the consistency of the indel calculation, we sequenced each PCR product from both sides of the cut (Table 1). In all cases, sequencing chromatograms already provided a clear visual impression of the presence of genetic modifications, mostly evidenced by a unique sequence before the cutting site and a mixture of sequences behind it (Additional file 1: Figure S6). In some cases, a small amount of mutated sequences was also detected before this cutting site, which likely corresponds to large indels that start after the sequencing primer (see arrows in Additional file 1: Figure S6). Strikingly, in cell lines enriched for *IRF3* and *p65* cleavage, no wt sequence of the respective genes was detected (Fig. 3a), while the amount of wt *TLR3* sequence was 8 to 36 %. In contrast, no indels were identified in non-enriched TLR3 gRNA-1* cells or in enriched *gfp* gRNA control cells (Fig. 3a, TLR3 gRNA-1 inset and upper plots). Therefore, this first approach indicated efficient enrichment of gene-edited cells. Interestingly, the mutation pattern was different for each tested gRNA and appeared to be of limited complexity, with a total of 4 to 11 indels for each gRNA (Fig. 3a and S7). This number may reflect a detection limit of the sequencing/TIDE approach. Nevertheless, as evidenced by the ranked frequency of indels, in most cases few indels represented the highest proportion of mutations (Additional file 1: Figure S7).

**Table 1:** Sequence of single guide RNA (gRNA), forward and reverse PCR primers (PCR-fw and PCR-rev), and primers used for sequencing (p1 and p2)

Gene	Type/Name	Sequence	Genomic location (Assembly Dec. 2013 GRCh38/hg38)
*IRF3* encoded on (−) strand of genome	gRNA-1	GCCACTGGTGCATATGTTCC	19:49663483–49663502 (−)
	gRNA-2	CCACTGGTGCATATGTTCCC	19:49663482–49663501 (−)
	gRNA-3	ATAAGCCAGACCTGCCAACC	19:49663455–49663474 (−)
	PCR-fw	TTCTCACCTGGGTATCAGAAGTA	19:49663181–49663203 (+)
	PCR-rev	TGAGGTTCCTAACTACCGAATTA	19:49664294–49664316 (−)
	p1	TGTCTGGCTGGGAAAAGTC	19:49663232–49663250 (+)
	p2	CTGTAATCCCAGCACTTTG	19:49663960–49663978 (−)
*TLR3* encoded on (+) strand of genome	gRNA-1	ACATTAGATCTGTCTCATAA	4:186078849–186078868 (+)
	gRNA-2	ATTAGGAACTCAGGTTCAGC	4:186078887–186078906 (+)
	gRNA-3	GGCTTGTCATCTACAAAATT	4:186078870–186078889 (+)
	PCR-fw	TGTGTTTGATAAGCCATGTGA	4:186078463–186078483 (+)
	PCR-rev	GGAGGCTAGAGAGGGAGAAC	4:186079465–186079484 (−)
	p1	AATCCTTCCTACAATGG	4:186078618–186078634 (+)
	p2	AGGATTGCTGGAAGACAGG	4:186079129–186079147 (−)
*p65* encoded on (−) strand of genome	gRNA-1	TCAATGGCTACACAGGACCA	11:65661815–65661834 (−)
	gRNA-2	AGGGACAGTGCGCATCTCCC	11:65661796–65661815 (−)
	gRNA-3	AGCTTGTAGGAAAGGACTGC	11:65661737–65661756 (−)
	PCR-fw	AATGGTTTTCTTCCTCAAACAA	11:65661392–65661413 (+)
	PCR-rev	CTTAGTTTCACCGCAGGTTCTA	11:65662331–65662352 (−)
	p1	GTATCCCCTGGAACTCATC	11:65661487–65661505 (+)
	p2	TGTTCCCCCTCATCTTC	11:65662186–65662202 (−)
*gfp*	gRNA	AAGGGCGAGGAGCTGTTCAC	-

Using the T7E1 assay, we obtained fractions of cleaved PCR product (cleaved/[cleaved + noncleaved]) ranging between 77 and 90 % (Fig. 3b and c, yellow bars). In contrast to the sequencing results, cells that were not treated with IZsCD95L still showed 41 % cleavage. The editing efficiency is typically calculated from the fraction of cleaved PCR products as follows [36]:

1 $$ \mathrm{editing}\ \mathrm{efficiency}=1-\sqrt{1- fractio{n}_{cleaved}} $$

Following this estimation, we obtained editing efficiencies ranging between 53 and 70 % for cells treated with IZsCD95L and 23 % for non-selected cells (Fig. 3d). Thus, both approaches verified successful editing and enrichment of cells. Yet, they showed a large discrepancy in the editing efficiency for enriched cells, reaching up to 100 % for the sequencing approach versus up to 70 % for the T7E1 approach. In contrast, non-enriched cells showed 0 % editing by sequencing but 23 % by the T7E1 approach (Fig. 3d, TLR3 gRNA-1*). These discrepancies led us to revisit the estimation of the editing efficiency. On the one hand, the Sanger sequencing might not be sensitive enough to detect a low fraction of mutant DNA species in the non-enriched sample, and vice versa, to detect a low amount of wt sequences in the enriched samples. On the other hand, the editing efficiency calculated using equation (1) relies on the assumption that the diversity of mutated strands is sufficiently large so that those mutated strands never reanneal with strands carrying the same mutation [36]. In other terms, only the wt strands would form homoduplexes and would remain uncleaved by the endonuclease. As we identified a low diversity of indels by sequencing, this questioned the validity of the aforementioned assumption. In order to test it, we calculated the expected cleavage fraction in an endonuclease assay by assuming that not only wt but also mutant homoduplexes can form. As explained in the Additional file 1: Supplemental note, this can be expressed as follows:

2 $$ fractio{n}_{cleaved}\approx fractio{n}_{heteroduplex}=1-{\displaystyle \sum_{i=1}^m{p}_j^2} $$

where m is the number of possible sequences, with m-1 mutants and one wt, and *p*_*j*_ is the relative amount of each of the sequences. As explained in more detail and exemplified in Additional file 1, this calculation shows that the cleavage fraction would not reach 100 % even when no wt sequence is present (Additional file 1: Figure S8). It also shows that for an editing efficiency below 30 %, the original equation (1) is particularly appropriate (see Additional file 1: Supplemental note) while for high editing efficiencies it becomes unsuitable.

To test the relevance of this calculation in practice, we hypothesized that the measured frequency of indels by the TIDE analysis mirrors the likelihood to obtain a mutation for a particular gRNA. Therefore, we applied equation (2) using the frequencies shown in Fig. 3a to derive the expected cleavage fraction in a T7E1 assay. Strikingly, the resulting predictions (Fig. 3c, green striped bars) were in good agreement with the measured cleavage fractions (Fig. 3c, yellow bars). We also estimated the endonuclease-mediated cleavage depending on varying amount of wt sequence (Additional file 1: Figure S9). By this means, we observed that the cleavage fraction cannot exceed 70 % to 90 % depending on the gRNA. Thus, the sensitivity of the Sanger sequencing and the aforementioned limitations of the interpretation of the T7E1 assay might readily explain the discrepancies in the estimation of the editing efficiency. Importantly, irrespective of the true editing efficiency, both approaches confirmed a strong enrichment of gene-edited cells when using CD95-induced apoptosis as selection strategy.

### Phenotyping of gene knock-out effects after death receptor-based enrichment

**Fig. 4:**
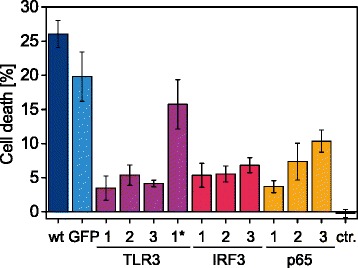
Poly (I:C)-induced cell death is reduced in cells with impaired TLR3, IRF3 and p65 expression. 24 h after transfection of 2 μg/ml Poly (I:C) with DOTAP, fractional cell death was measured by flow cytometry using propidium iodide. We compared wt Hela cells and polyclonal HeLa cell lines generated with the nine different gRNAs targeting *TLR3*, *IRF3* and *p65* genes and the control gRNA targeting GFP. *TLR3* gRNA-1 cells denoted with a star (*) were not treated with IZsCD95L, while *ctr.* denotes HeLa wt cells transfected with a non-triphosphate 19-mer RNA control to account for cell death due to transfection with DOTAP. Shown are means ± s.e.m. of 3 independent experiments

As a proof-of-concept, we used the different HeLa cell lines for a direct phenotyping of introduced gene mutations. Specifically, we were interested in the role of p65, IRF3 and TLR3 in double-stranded (ds)RNA-induced apoptosis. Some previous studies reported the requirement of transcriptional activity through p65 and/or IRF3 for this type of death [37–40], while others showed the possibility of direct cell death through the formation of the ripoptosome on activated TLR3 [41, 42]. Notably, this cell death response typically only occurred in a fraction of the cell population [41, 42]. To investigate the role of the three genes in dsRNA-induced death in HeLa cells, we quantified the death response of edited cells after Poly (I:C) treatment by flow cytometry. Using wt HeLa cells and *gfp* gRNA-treated cells as controls, we obtained a cell death of 26.0 ± 2.0 and 19.8 ± 3.6 %, respectively. Interestingly, in *p65*, *IRF3* and *TLR3* gene-edited cell lines, the cell death response was strongly reduced, ranging between 3.4 ± 1.8 with *TLR3* gRNA-1 and 10.3 ± 1.6 % with *p65* gRNA-3 (Fig. 4). Notably, still 15.7 ± 3.6 % of non-enriched *TLR3* gRNA-1* cells were killed by Poly (I:C) treatment. Together, this emphasizes that the three genes are involved in this type of death and that enrichment of gene-edited cells helps to directly assess the associated cellular phenotype.

### *Comparison of cell enrichment by death receptors* versus *puromycin*

Finally, we compared the gene editing efficiency in HeLa, HT-1080 and LN-18 cells when using the death receptor-based versus the puromycin-based selection approach. For this, we targeted the gene *IRF3* using gRNA-3 either with the Cas9–2a-CD95ΔDD plasmid used above, as CD95L was most potent to kill those cell lines, or with the Cas9–2a-puro plasmid (shown in Fig. 1a). Two days after transfection with Cas9–2a-CD95ΔDD, cells were incubated with IZsCD95L for 5 h or left untreated. HeLa, HT-1080 and LN-18 cells transfected with Cas9–2a-puro were treated for 30 h with 5, 3 and 5 μg/ml puromycin, respectively, or left untreated. The concentrations were chosen to ensure a complete killing of the cells (Fig. 2c and S10a). Cells were then maintained in culture until the density of all treated cells allowed their characterization, between 7 and 11 days post-transfection.

**Fig. 5:**
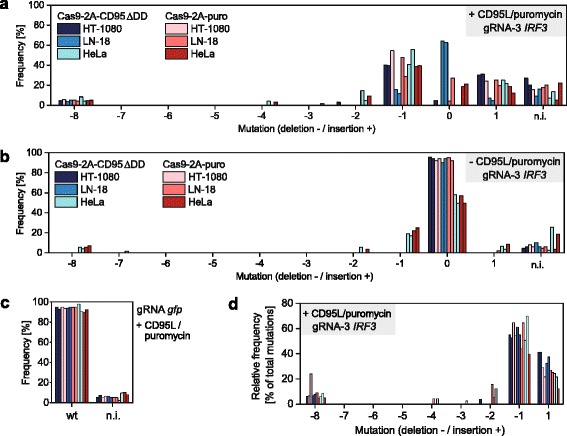
Death receptor-based and puromycin-based enrichment of Cas9-expressing cells. In two independent experiments, HT-1080, LN-18 and HeLa cells were transfected with plasmid Cas9-2A-CD95∆DD or Cas9-2A-puro encoding gRNA-3 targeting the *IRF3* gene or gRNA targeting *gfp*. Cell lines were tested against wild type cells for presence of indels in the gene *IRF3* using primer IRF3 p1 (Table 1). Indels were detected by Sanger sequencing and TIDE analysis. **a** Selection using IZsCD95L or 5 μg/ml puromycin treatment. **b** No selection. **c** *gfp* gRNA cells treated with IZsCD95L or 5 μg/ml puromycin. Mutatio*n* = 0 represents wild type sequence. The non-interpretable fraction (n.i.) relates to the correlation coefficient with R^2^ = 100 - n.i. **d** The relative frequency of indels in enriched cells was calculated by dividing their amplitude from panel 5a by the sum of all indels

As assessed by the TIDE analysis of sequenced PCR products, we obtained different efficiencies depending on the cell type and the selection strategy (Fig. 5). For each condition, the fraction of indels was calculated by dividing their amount by the amount of wt and indel sequences (Fig. 5a). For death receptor-enriched cells, the fraction of indels in HT-1080 cells was 100 and 94 %, in LN-18 cells 29 and 25 %, and in HeLa cells 100 and 100 %, respectively, in two biological replicates each. For puromycin-enriched cells, HT-1080 cells showed 100 % indels, LN-18 cells 94 and 65 %, and HeLa cells 80 and 73 %. In contrast, in non-enriched transfected cells, indels were detected only in one replicate of LN-18 cells transfected with Cas9–2a-puro (2.2 %), in HeLa cells with Cas9–2a-puro (41 and 39 %), and in HeLa cells with Cas9–2a-CD95ΔDD (40 and 33 %) (Fig. 5b). As expected, enriched and transfected control cells (*gfp* gRNA) displayed only wt sequences (Fig. 5c). Finally, Western blotting confirmed the different observed efficiencies at the protein level, with equal efficiencies for both strategies in HT-1080 cells, a slight advantage for puromycin-selected LN-18 cells, and better efficiencies for death receptor-selected HeLa cells (Additional file 1: Figure S10b). Notably, as in the pilot experiment (Fig. 3), we found the frequent deletion of 1 and 8 nucleotides as well as insertion of 1 nucleotide with this gRNA (*IRF3* gRNA-3). Remarkably, in each cell line and in each replicate, their relative frequency was similar (Fig. 5d).

## Discussion

The CRISPR/Cas9 technology allows for the study of gene function through targeted DNA modification. In principle, it permits the investigation of cellular networks with a greater sensitivity than with RNA interference approaches where gene expression reduction is only partial. However, this sensitivity can be tempered by the limited delivery efficiency of Cas9 and the gRNA [3, 6, 15]. Different strategies were employed to increase the percentage of Cas9-expressing or gene-edited cells: isolation of single cells to generate monoclonal cell lines or generation of polyclonal cells by enriching Cas9-expressing cells through GFP fluorescence or antibiotics like puromycin [20] or magnetic bead isolation of receptor-overexpressing cells [24]. In this work, we extended this panel of options with the death receptor-based Cas9 selection strategy. Similar to antibiotics-based selection, our new strategy does not require any transfer of the cells during the selection process. Moreover, our method can be applied in cell lines that already stably express resistance genes. Importantly, while the selection efficiency matches the one obtained with puromycin, the death receptor-based method minimizes the time required for the selection. Finally, it can be used in laboratories with no capacity to sort cells or to produce viruses. One drawback is the applicability to cells that are fully death ligand-sensitive. This is for example not the case for induced pluripotent stem cells [43], and testing of the effect of the ligand on cells of interest should be the first step to be performed. However, the production of the death ligand can be easily accomplished in the laboratory, does not require protein purification and is inexpensive.

Thus far, the CRISPR/Cas9 technology has been applied to pooled genomic screens, where upon negative or positive selection, gene hits are identified by sequencing of the stably integrated gRNA [7]. We envision a major application of our death receptor-based enrichment strategy in arrayed screens, where, as an alternative option to antibiotic-based enrichment, it should increase the sensitivity of phenotyping. In this context, cell transfection, selection, washing and expansion could be implemented in the same plate prior to phenotyping. Hence, existing pipelines for arrayed RNAi screens could be easily adapted [14]. Parameters to be optimized would be the cell number to be plated and the time for their expansion, so that the cell density allows for phenotyping. Here, we have exemplarily shown the involvement of three genes in Poly (I:C)-induced cell death in HeLa cells, and we plan to use this array approach to identify additional genes involved in this as well as other pathways induced by dsRNA. Notably, thanks to the power of the enrichment approach, optimization of the transfection efficiency was not required.

In order to quantify the efficiency of gene editing by the CRISPR/Cas9 method, we employed and compared the T7E1 assay and the TIDE analysis [35]. Although both approaches yielded congruent results on the enrichment of gene editing after death receptor-mediated cell killing, they generated significantly different outputs for the editing efficiency. As explained in the results and in the Additional file 1: Supplemental note, the estimate of the editing efficiency calculated from an endonuclease assay result is strongly dependent on the underlying assumptions. While equation (1) relies on the postulation that mutated strands cannot anneal with strands carrying the same mutation due to a hypothetically large diversity of indels, the more general equation (2) shows that equation (1) is actually only valid under two conditions: low editing efficiency and/or very large diversity of indels all exhibiting low probabilities. In this work, we obtained high editing efficiencies, with mostly few indels representing the majority of mutations (Additional file 1: Figure S7). Hence, equation (2) should be more appropriate compared to equation (1) traditionally used to assess genome editing efficiency. Confirming its relevance, the symmetric parabolic shape obtained from this equation with the most extreme case of one possible indel (Additional file 1: Figure S8) has been observed in an in vitro experiment that mimics this situation [44]. Simulation of such situations led to several conclusions that are of general interest when interpreting the cleavage fraction (fraction_cleaved_ = cleaved/[cleaved + noncleaved]) from the T7E1 or Surveyor assay to determine editing efficiency:

1. One cannot reach a 100 % cleavage fraction with the T7E1 assay, even in perfect technical setups.
2. Due to the parabolic shape of equation (2), within a certain range, two possible editing efficiencies can be assigned to one cleavage fraction (Additional file 1: Figure S8).
3. The maximal cleavage fraction does not correspond to the maximal editing efficiency.
4. Although equation (2) is universal, its coefficients depend on the indel pattern, not known a priori.
5. When editing efficiency is high, the sensitivity of the T7E1 assay to detect changes in this efficiency is particularly poor. Although this effect can also be seen for equation (1) for high editing efficiencies, it becomes even stronger when one takes into account the limited diversity of indels with equation (2) (Additional file 1: Figure S8).
6. For a low editing efficiency, the relationship between cleavage and efficiency is reasonably independent of the indel pattern, and the T7E1 assay is particularly sensitive (see Additional file 1: Supplemental note for details). Therefore, comparison of different gRNAs, which generate different patterns, should be performed under conditions with more than 70 % wt sequence.

On top of these theoretical considerations, one should mention that the T7E1 and Surveyor assays are likely unable to generate a complete cleavage of duplexes. A direct comparison of both assays indicated a maximum of 80 and 60 % cleavage of heteroduplexes with the T7E1 and Surveyor assays, respectively [44]. In this work, by taking into account the different indels that were identified from the TIDE analysis for the distinct gRNAs used in this study, we observed that the range of cleaved fraction using the T7E1 assay, between 75 and 90 %, was theoretically compatible with a 100 % editing efficiency. In practice, however, this means that when large amounts of cleaved PCR products are observed by T7E1, a precise estimation of editing efficiency using this assay cannot be expected.

Overall, the sequencing approach provided not only information on editing efficiency, but also interesting information on the type and relative amount of indels. Since the indel pattern using *IRF3* gRNA-3 was reproduced in different cell lines and experiments, our data strengthen the notion that the type of indel is governed by the choice of the gRNA sequence. Moreover, as *IRF3* gRNA-1 and gRNA-2 were only shifted by 1 nucleotide but showed a very different indel pattern, the cutting position is likely to have a strong influence on this pattern. DNA repair studies suggest that this pattern is driven by the presence of microhomology sequences on both sides of the cut. Such sequences of 1 to 4 nucleotides can influence the repair by non-homologous end joining [45], while larger ones may lead to microhomology-mediated end joining even if they are further apart from the cutting site [46–48]. A closer examination of the gRNA sequences (Additional file 1: Figure S11) allowed to identify three microhomology domains that may explain prominent peaks for deletion of 6, 8 and 6 nucleotides for *TLR3* gRNA-2, *IRF3* gRNA-3 and *p65* gRNA-1, respectively. The use of computer tools that predict indels using microhomology sequences like the one presented in reference [48] should therefore help to design gRNAs that generate interesting indel patterns.

## Conclusions

Our work demonstrates the usefulness of death receptors as tool for the selection of Cas9-expressing cells. Killing non-transfected cells with death ligands can trigger enrichment of gene-edited cells that allows for direct assessment of gene function. The here presented extension of the tool panel based on the CRISPR/Cas9 technology will particularly enhance low- and high-throughput phenotyping screening after CRISPR-mediated gene knockdown.

## Methods

### Cell culture

MDA-MB-231 and HT1080 cells were obtained from CLS (Eppelheim, Germany). LN-18 cells were a kind gift from Ana Martin-Villalba. MCF-10A cells were obtained from ATCC (Manassas, VA, USA). HeLa cells were the so-called HeLa Kyoto and were obtained from Holger Erfle. A549 and 293 T cells were kind gifts from Ralf Bartenschlager and Dirk Grimm, respectively. HeLa, HT-1080, MDA-MB-231, LN-18, 293 T and A549 cells were maintained in Dulbecco’s modified eagle medium (DMEM, Life Technologies, Darmstadt, Germany) containing 10 % fetal calf serum (Biochrom AG, Berlin, Germany), penicillin/streptomycin (100 μg/ml each, Life Technologies). MCF 10A cells were maintained in DMEM with F12 (Life Technologies), 5 % horse serum (Biochrom AG), 20 ng/ml EGF (TEBU-Bio, Offenbach, Germany), 0.5 μg/ml hydrocortisone (Sigma-Aldrich, St. Louis, Missouri, USA), 10 μg/ml insulin (Sigma-Aldrich) and penicillin/streptomycin (100 μg/ml each, Invitrogen). For ligand production, 293 T cells were transfected using branched polyethylenimine (PEIpro, PolyPlus, Illkirch, France). In detail, for 20 ml of ligand, 4.5 million cells were plated in a 15 cm cell culture dish, transfected the next day with 12 μg of plasmid and washed 24 h later. The supernatant was collected 48 h after washing, and stored at 4 °C for several months. The concentration was typically in the range of 1 μg/ml as estimated by quantitative western blotting (data not shown). X-tremeGENE was used for the transfection of the Cas9 constructs, while Polyinosinic:polycytidylic acid (Poly(I:C)) and 5’ppp-dsRNA control (Invivogen, San Diego, CA, USA) were transfected with DOTAP (Carl Roth, Karlsruhe, Germany). Puromycin was from Life Technologies.

### Plasmids

DR4-ΔDD and DR5-ΔDD were amplified from Gateway full ORF clones from the genomics and proteomics core facilities of the DKFZ, Heidelberg. CD95-ΔDD was amplified from the full-length coding sequence of CD95 kindly provided by Peter Krammer. DR4-ΔDD, DR5-ΔDD and CD95-ΔDD contain the first 290, 250 and 210 amino acids of the full-length human DR4, DR5 and CD95, respectively. The gene coding for puromycin *N*-acetyl-transferase was amplified from the Cas9–2a-puro plasmid used in Shalem et al. [10] (originally Addgene #49535). The Cas9 sequence is the one derived from *Streptococcus pyogenes* originally used in Shalem et al. [10] (Addgene #49535). It is preceded by a CMV promoter, and followed by a SV40 nuclear localization sequence, the 2A peptide from *Thosea asigna* virus T2A EGRGSLLTCGDVEENPGP, a peptide linker RSMH and the cDNA of one of the four resistance genes without the first methionine-encoding triplet. In the Cas9-T2A-DR5ΔDD-F2A-DR4ΔDD construct, the DR5ΔDD and DR4ΔDD are separated by the *foot-and-mouth disease virus* 2A peptide F2A VKQTLNFDLLKLAGDVESNPGP preceded by the linker SG and followed by the linker RSMH. In construct Cas9-T2A-DR5ΔDD-F2A-DR4ΔDD-P2A-CD95ΔDD, the additional CD95ΔDD is separated from DR4ΔDD by the *porcine teschovirus-1* 2A peptide P2A ATNFSLLKQAGDVEENPGP, and preceded by the linker GSG and followed by the linker RSMH. The coding sequence for the guide RNA was cloned on the same plasmid and driven by a 7SK promoter. gRNA design was performed using the ZiFiT Targeter software [49, 50]. All constructs were cloned based on the pSSV9 vector [51], in which the entire cassette consisting of the Cas9 and gRNA expression units was inserted between the two XbaI sites.

IZsCD95L [33], kindly provided by Henning Walczak, was cloned into pIRES-puro2 (Clontech Laboratories, Inc., CA, USA), and IZsTRAIL was built from IZsCD95L in pIRES-puro2. In the following the amino acid sequence for IZsCD95L and IZsTRAIL in one-letter code starting from the N-terminus is given. To allow protein secretion, we used the N-terminal signal sequence MGTPHLQGFLLLFPLLLRLHGASAGS in the construct IZsTRAIL, and the signal sequence MARRLWILSLLAVTLTVALAALE in the construct IZsCD95L. In both constructs, those were followed by the Flag tag DYKDDDDK and amino acids PSQKSKRRTSSDRMKQIEDKIEEILSKIYHIENEIARIKKLIGERTR encoding the isoleucine-zipper domain (PDB entry 1GCM). In IZsCD95L, the sequence encoding CD95L from amino acids 117–281 (STSQ…LYKL) was used. In IZsTRAIL, the linker SGGSSG bridges the IZ domain to the extracellular receptor binding domain of TRAIL from amino acids 122–281 (VAAH…FLVG). The sequence of TRAIL was amplified by PCR from cDNA of NK92-C1 cells, kindly provided by Carsten Watzl.

### Western blot

Western blots were performed by lysing cells using ice-cooled lysis buffer (20 mM Tris/HCl, pH 7.5, 150 mM NaCl, 1 mM phenylmethylsulfonyl fluoride (Sigma-Aldrich), protease inhibitor cocktail, 1 Triton X-100 (Serva, Mannheim, Germany), and 10 % glycerol). Lysates were analyzed using SDS-PAGE gels (Novex NuPAGE 10 % Bis-Tris Protein Gel, Life Technologies). Proteins were transferred to PVDF membrane (Millipore, Billerica, MA, USA) using wet blotting. IRF3 was detected using the primary antibody clone D83B9 (Cell Signaling Technology, Danvers, MA, USA). Secondary anti-rabbit antibodies were HRP-conjugated (Dianova, Hamburg, Germany). Detection was performed using the Pico Chemiluminescent Substrate from Thermo Scientific (Asheville, NC, USA) and a CCD camera.

### Immunofluorescence

CD95, DR4 and DR5 were detected in living cells using the antibody clones DX2, DJR1 and DJR2–4, respectively, each labeled with phycoerythrin (PE) (eBioscience Inc, San Diego, CA, USA). Cells were trypsinized, blocked on ice with 1 % bovine serum albumin (BSA, Sigma) in PBS, incubated for 30 min with antibody in blocking buffer on ice and directly measured by flow cytometry (Beckman Coulter, Krefeld, Germany).

### Poly (I:C) treatment

75000 cells were seeded into wells of a 24-well plate and treated one day later by transfection of 2 μg/ml (final concentration, f.c.) Poly (I:C). 24 h later, dead and living cells were collected. Cell supernatant, potentially containing living and dead cells, was transferred into empty wells of a 24-well plate. Remaining cells were washed once with PBS and trypsinized. PBS and trypsinized cells were merged with the previously collected cell supernatant. Sample was incubated with propidium iodide (solution, 1 μg/ml f.c., Sigma-Aldrich) 10 min before measurement by flow cytometry (Beckman Coulter).

### Editing analysis

Genomic DNA was extracted using the DirectPCR® Lysis-Regent Cell (Peqlab, Erlangen, Germany) according to the protocol. PCRs were performed using the Phusion Flash PCR master mix (Thermo Scientific). T7 endonuclease I was from NEB (Ipswich, MA, USA). Oligonucleotides were from Eurofins MWG Operon (Ebersberg, Germany), and Sanger sequencing was performed by GATC Biotech (Konstanz, Germany).

### Microscopy

Cell death was measured by microscopy using transmission imaging and visual cell morphology identification. Images were taken on a Leica SP5 confocal microscope (Leica Microsystems, Mannheim, Germany) or on an Olympus CKX41 wide-field microscope equipped with an Olympus PEN Lite CCD camera (Olympus Europa, Hamburg, Germany). Cell death kinetics were quantified from images by manually marking the first rounding and shrinkage event due to cell death. Marks were then automatically segmented and counted in ImageJ.

### Computer simulations

Simulations to predict the T7E1 cleavage fraction from indel patterns were performed using MATLAB (The MathWorks GmbH, Ismaning, Germany).

### Ethics

No ethics approval was required for use of any of the cell lines in this study. Cell lines used in this study were MDA-MB-231, HT1080, LN-18, MCF-10A, HeLa, A549 and 293 T cells and are available from the ATCC organization (Manassas, VA, USA).

## Additional file

Additional file 1: Figure S1. Transient expression of death receptor mutants in HeLa cells using Cas9-2A constructs was quantified by flow cytometry. **Figure S2.** Transient expression of death receptor mutants in HT-1080 cells using Cas9-2A constructs was quantified by flow cytometry, as in Figure S2. **Figure S3.** Transient expression of death receptor mutants in LN-18 cells using Cas9-2A constructs was quantified by flow cytometry, as in Figure S2. **Figure S4.** Transient expression of death receptor mutants in MDA-MB-231 cells using Cas9-2A constructs was quantified by flow cytometry, as in Figure S2. **Figure S5.** Cell death kinetics of MDA-MB-231 using 1 and 3 μg/ml puromycin (*n* = 181 and 182 cells) and of LN-18 cells using 3 and 5 μg/ml puromycin (*n* = 149 and 118 cells) measured by microscopy. **Figure S6.** Plots were generated using the software TIDE (http://tide.nki.nl/) and modified for clarity. **Figure S7.** Ranked frequency of indels, normalized to 100% for the total (upper plot) or for the most frequent indel (lower plot). **Figure S8.** Estimate of the cleavage fraction obtained by the T7E1 assay. **Figure S9.** Estimate of endonuclease mediated cleavage depending on the amount of wild type for experimentally observed indel patterns. **Figure S10.** Death receptor-based and puromycin-based enrichment of Cas-9 expressing cells. **Figure S11.** The TIDE analysis provided information on the insertions and deletions that are introduced in a specific genomic region targeted by the gRNAs. **Supplemental Note** - Editing Efficiency. (PDF 5416 kb)

## Abbreviations

AAV: adeno-associated virus
CRISPR: clustered regularly interspaced short palindromic repeats
DD: death domain
DR: death receptor
gRNA: single guide RNA
indel: insertion/deletion
T7E1: T7 endonuclease I
TIDE: Tracking of Indels by DEcomposition
wt: wild type
