# Supplementary material for: Death receptor-based enrichment of Cas9-expressing cells
Source: BMC Biotechnol. 2016 Feb 16;16:17. doi: 10.1186/s12896-016-0250-4 (PMC4755021; doi:10.1186/s12896-016-0250-4)
Supplement: Additional file 1: Figure S1. — Transient expression of death receptor mutants in HeLa cells using Cas9-2A constructs was quantified by flow cytometry. Figure S2. Transient expression of death receptor mutants in HT-1080 cells using Cas9-2A constructs was quantified by flow cytometry, as in Figure S2. Figure S3. Transient expression of death receptor mutants in LN-18 cells using Cas9-2A constructs was quantified by flow cytometry, as in Figure S2. Figure S4. Transient expression of death receptor mutants in MDA-MB-231 cells using Cas9-2A constructs was quantified by flow cytometry, as in Figure S2. Figure S5. Cell death kinetics of MDA-MB-231 using 1 and 3 μg/ml puromycin (n = 181 and 182 cells) and of LN-18 cells using 3 and 5 μg/ml puromycin (n = 149 and 118 cells) measured by microscopy. Figure S6. Plots were generated using the software TIDE (http://tide.nki.nl/) and modified for clarity. Figure S7. Ranked frequency of indels, normalized to 100% for the total (upper plot) or for the most frequent indel (lower plot). Figure S8. Estimate of the cleavage fraction obtained by the T7E1 assay. Figure S9. Estimate of endonuclease mediated cleavage depending on the amount of wild type for experimentally observed indel patterns. Figure S10. Death receptor-based and puromycin-based enrichment of Cas-9 expressing cells. Figure S11. The TIDE analysis provided information on the insertions and deletions that are introduced in a specific genomic region targeted by the gRNAs. Supplemental Note - Editing Efficiency. (PDF 5416 kb) [file 12896_2016_250_MOESM1_ESM.pdf]

Death receptor-based enrichment of Cas9-expressing cells

C. Liesche, L. Venkatraman, S. Aschenbrenner, S. Grosse, D. Grimm, R. Eils and J. Beaudouin

Supplemental Material

Expression of death receptor mutants: HeLa . . . 2
Expression of death receptor mutants: HT-1080 . . . 3
Expression of death receptor mutants: LN-18 . . . 4
Expression of death receptor mutants: MDA-MB-231 . . . 5
Puromycin cell death kinetics . . . 6
Sequencing results of HeLa cell lines: IRF3, TLR3 and p65 . . . 7
Frequency of indels . . . 8
Estimating the cleavage fraction assuming homoduplex formation of mutant DNA . . . 9
Estimate of the cleavage fraction using Sanger sequencing data . . . 10
Death receptor-based and puromycin-based enrichment of Cas-9 expressing cells . . . 11
Homology domains . . . 12

Supplemental Note

Estimating the editing efficiency assuming formation of homoduplexes of mutant DNA . . . 13

Supplemental Figure 1

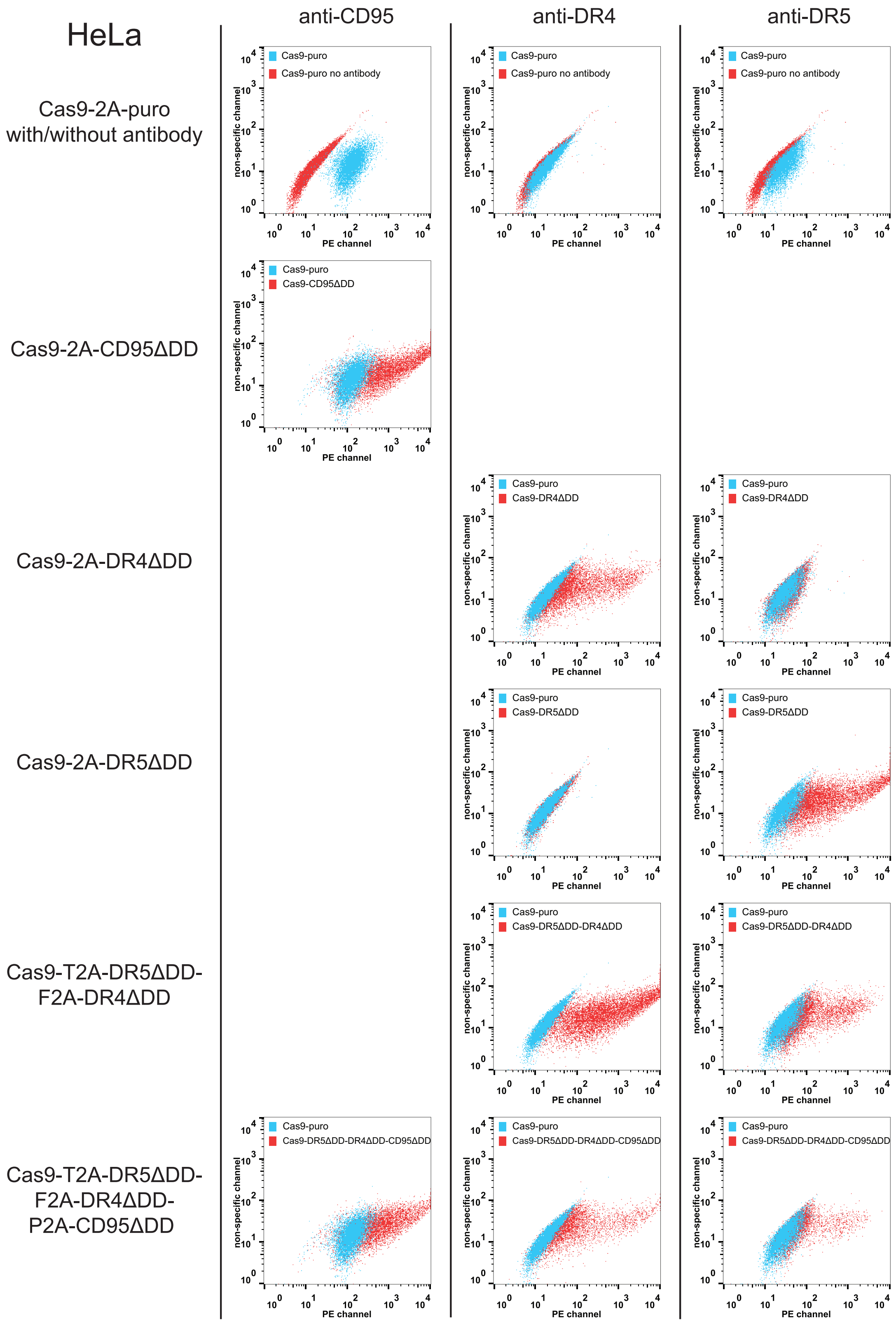

Figure S 1: Transient expression of death receptor mutants in HeLa cells using Cas9-2A constructs was quantified by flow cytometry. Phycoerythrin (PE)-labeled antibodies were used. Cas9 nuclease was co-expressed with one of the truncated death receptors or with puromycin as control (Figure 1b main text). The first row shows staining of cells transfected with Cas9-2A-puro to assess endogenous levels of receptor expression. Additional controls are Cas9-2A-DR4ΔDD stained with anti-DR5 and Cas9-2A-DR5ΔDD stained with anti-DR4 (third and fourth row) . These data show that overexpression of the different death receptor truncations using the Cas9-2A-constructs can be achieved.

Supplemental Figure 2

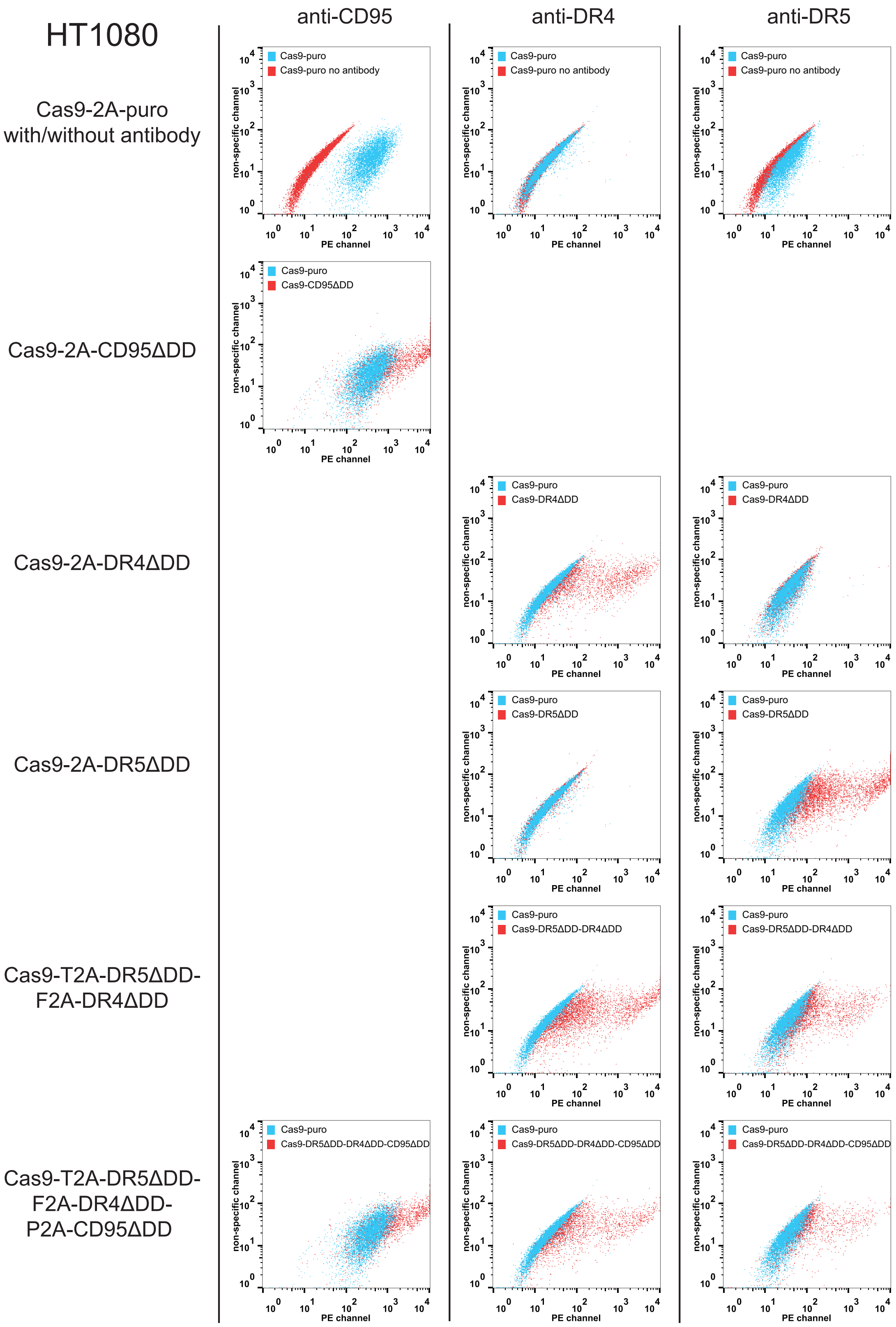

Figure S 2: Transient expression of death receptor mutants in HT-1080 cells using Cas9-2A constructs was quantified by flow cytometry, as in Figure S 2.

Supplemental Figure 3

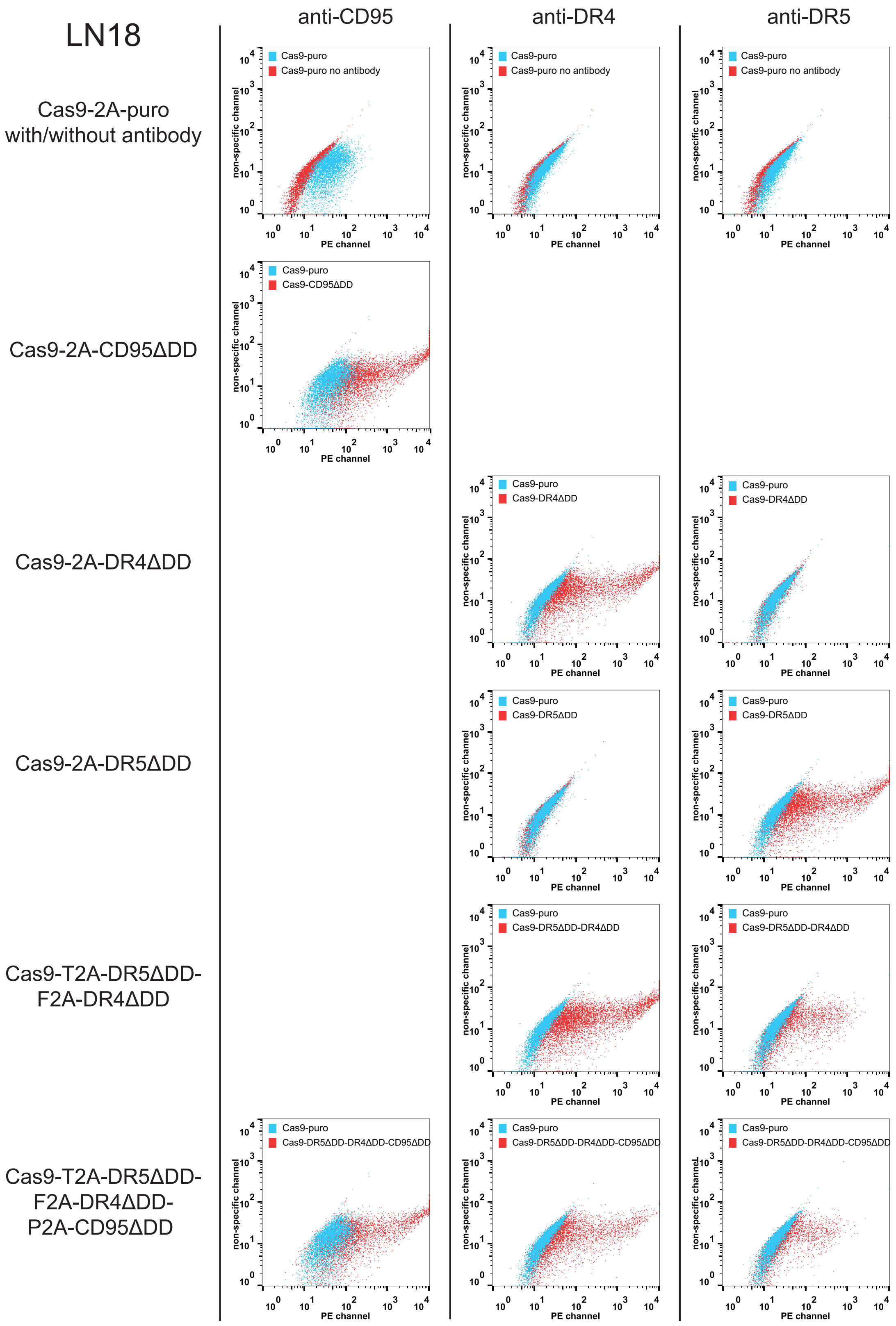

Figure S 3: Transient expression of death receptor mutants in LN-18 cells using Cas9-2A constructs was quantified by flow cytometry, as in Figure S 2.

Supplemental Figure 4

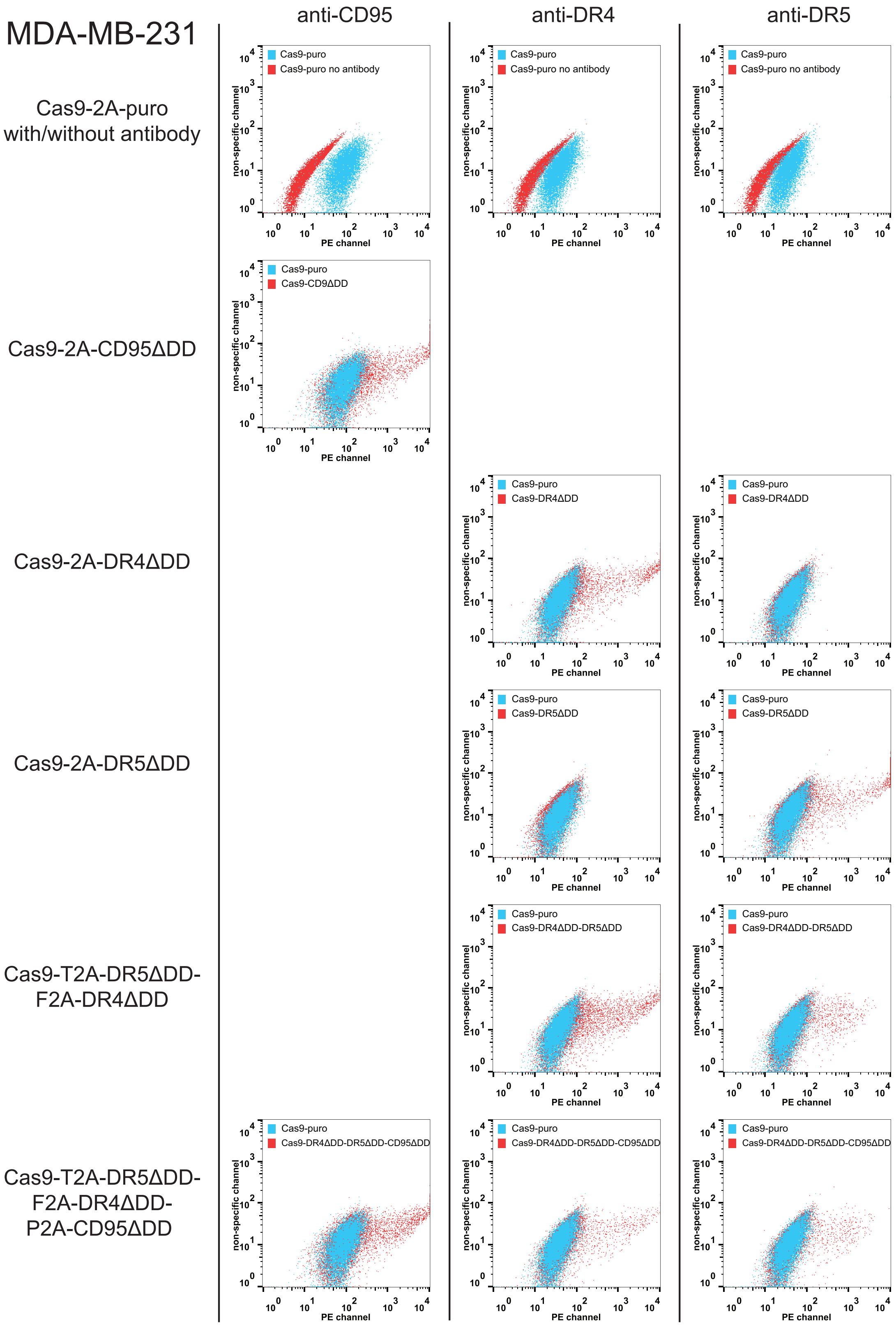

Figure S 4: Transient expression of death receptor mutants in MDA-MB-231 cells using Cas9-2A constructs was quantified by flow cytometry, as in Figure S 2.

Supplemental Figure 5

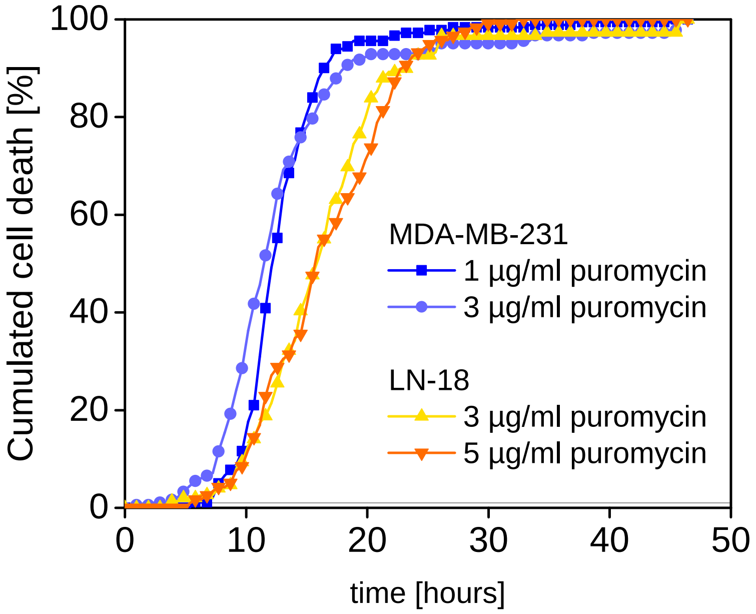

Figure S 5: Cell death kinetics of MDA-MB-231 using 1 and 3 µg/ml puromycin (n = 181 and 182 cells) and of LN-18 cells using 3 and 5 µg/ml puromycin (n = 149 and 118 cells) measured by microscopy. Cell death kinetics were saturated at these concentrations of puromycin. Images were taken every 30 minutes.

Supplemental Figure 6

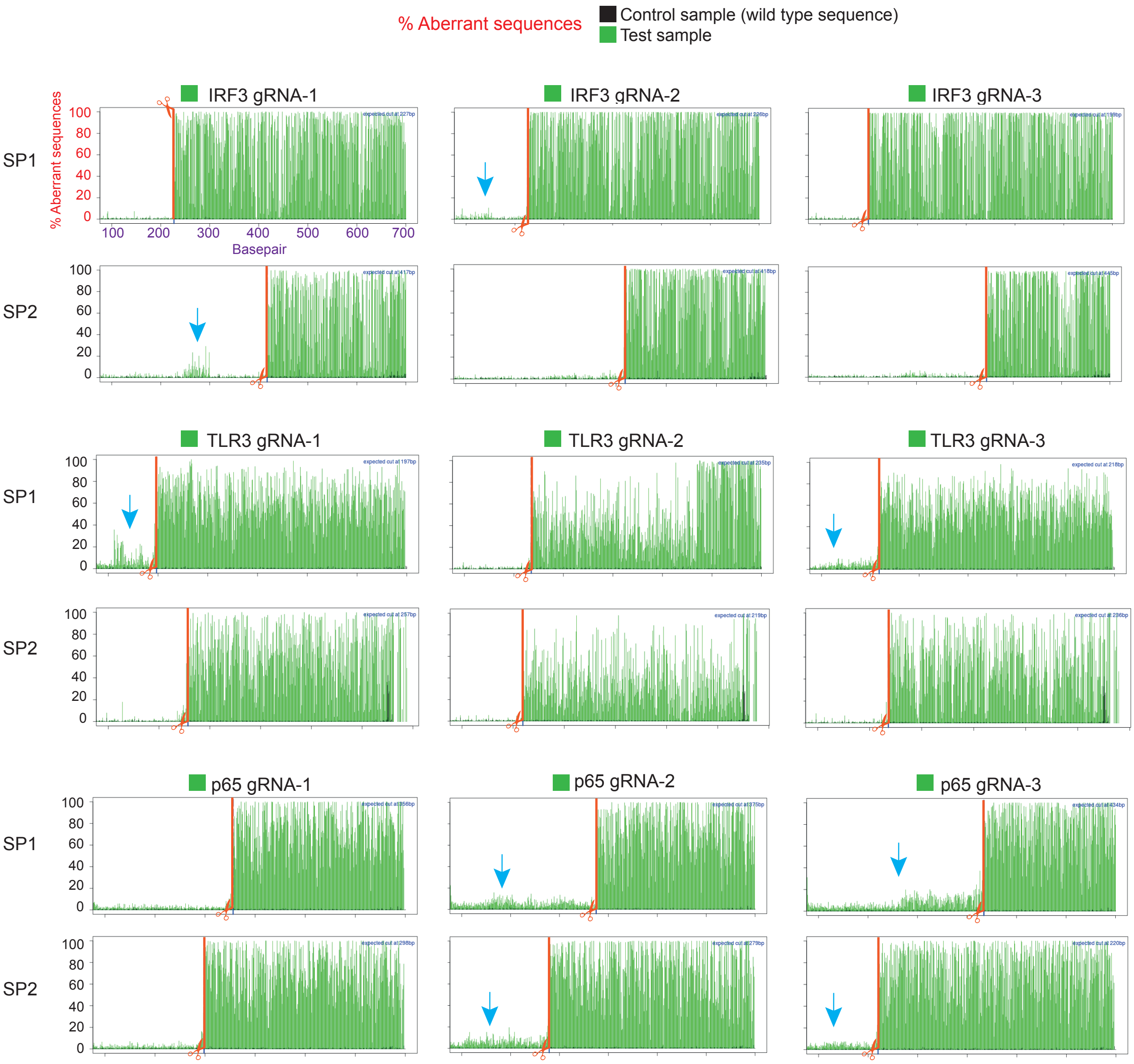

Figure S 6: Plots were generated using the software TIDE (http://tide.nki.nl/) and modified for clarity. Plots show the percentage of aberrant sequences along the sequence of the PCR products. The sequencing result from wild type cells served as reference. For each polyclonal cell line, made by different gRNAs, two sequencing primers (denoted SP1 and SP2) were used. The percentage of aberrant sequences increases sharply after the nuclease cleavage site (orange line). Arrows point towards aberrant sequence likely corresponding to larger indels or more complex mutations.

Supplemental Figure 7

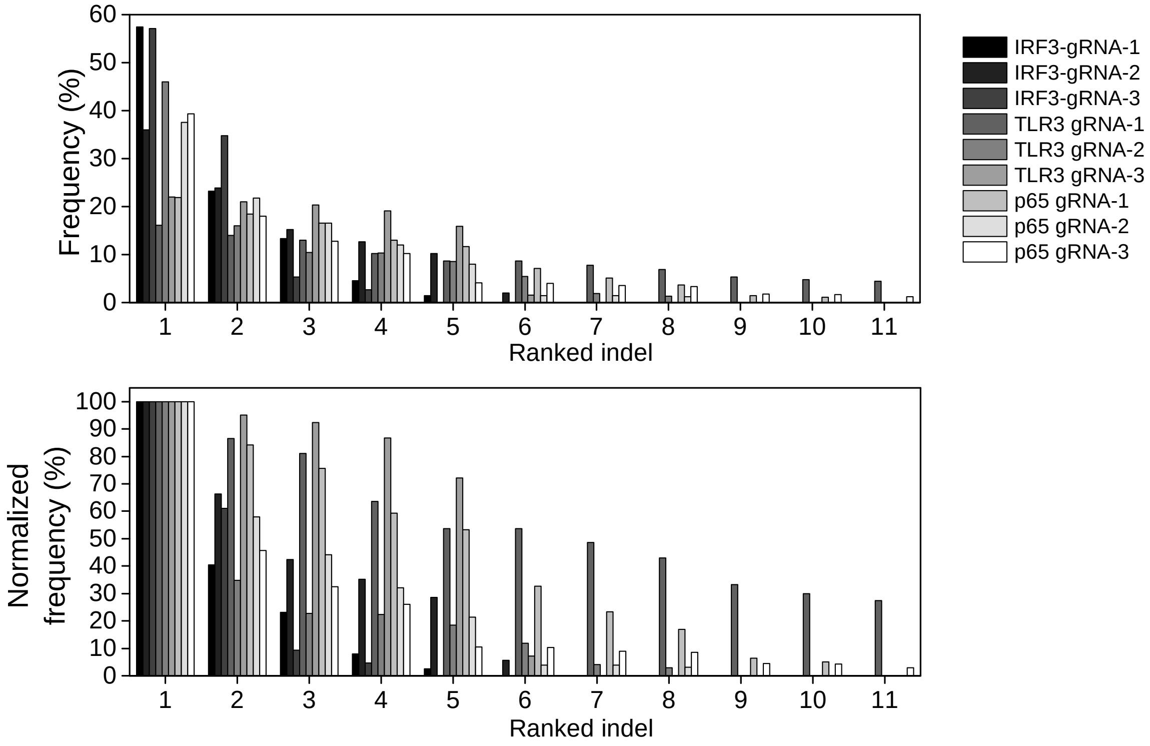

Figure S 7: Ranked frequency of indels, normalized to 100% for the total (upper plot) or for the most frequent indel (lower plot). The Sanger sequencing and TIDE analysis generated different indel patterns for the different gRNA. For each indel, we took the mean of the frequency values obtained from the different sequencing and ranked them. Few indels represented the highest proportion, while the case of *TLR3* gRNA-1 rather represents an exception with 11 indels of significant frequency. These data illustrate that homoduplex formation of mutated strands is likely when performing an T7E1 assay with mammalian cells.

Supplemental Figure 8

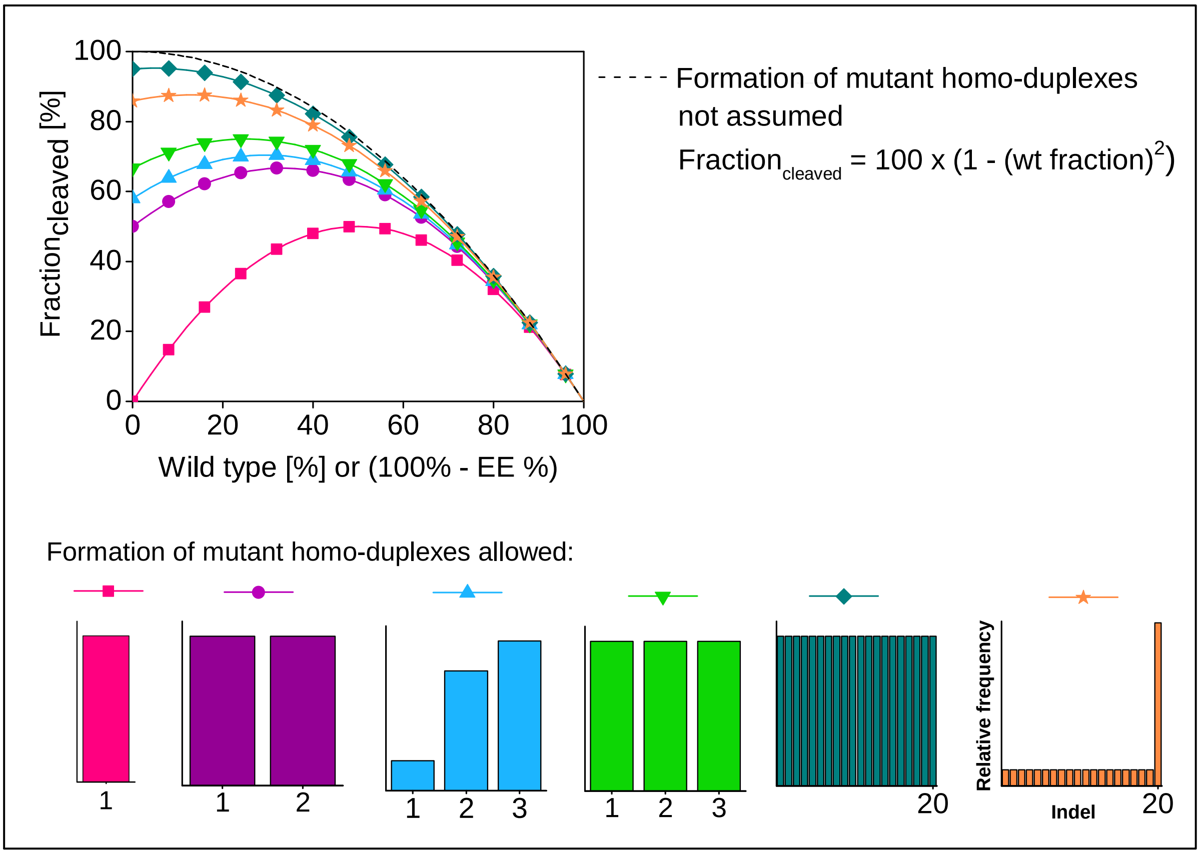

Figure S 8: Estimate of the cleavage fraction obtained by the T7E1 assay. The editing efficiency EE relates to the fraction of wild type with $EE = 100 - fraction_{wildtype}$. The line graph shows the predicted cleavage depending on the amount of wild type sequence present in the sample. Assuming the model using equation (1) (dashed line), a unique solution of the amount of wild type for a given cleavage fraction exists. When wild type sequence is absent, this equation predicts that cleavage can reach 100%. However, this equation relies on the absence of homoduplexes formed by mutated strands, which would require an extremely large amount of possible indels all with low probability. In contrast, assuming a model that allows the formation of mutant homoduplexes (solid lines), the predicted cleavage cannot reach 100%. Moreover, for cleavage fractions ranging from their value at 0% wild type to their maximum, two editing efficiency values can correspond to one cleavage fraction.

The different solid line graphs were calculated assuming different pattern of indels. Those different patterns are illustrated in the bar diagrams below, with 1 to 20 indels and varying probabilities/frequencies. The lines get closer to the dashed line with increasing indel numbers of equal probability. For identical indel numbers, unequal probabilities reduce the cleavage fraction (compare lines with triangles light blue and green or lines with diamonds and stars). For more than one possible indel, one can also note that the cleavage fraction varies weakly with the wild type percentage when that one is small. On the contrary, when the wild type percentage is high, the dependence on the indel pattern disappears and equation (1) provides a good prediction of editing efficiency.

Supplemental Figure 9

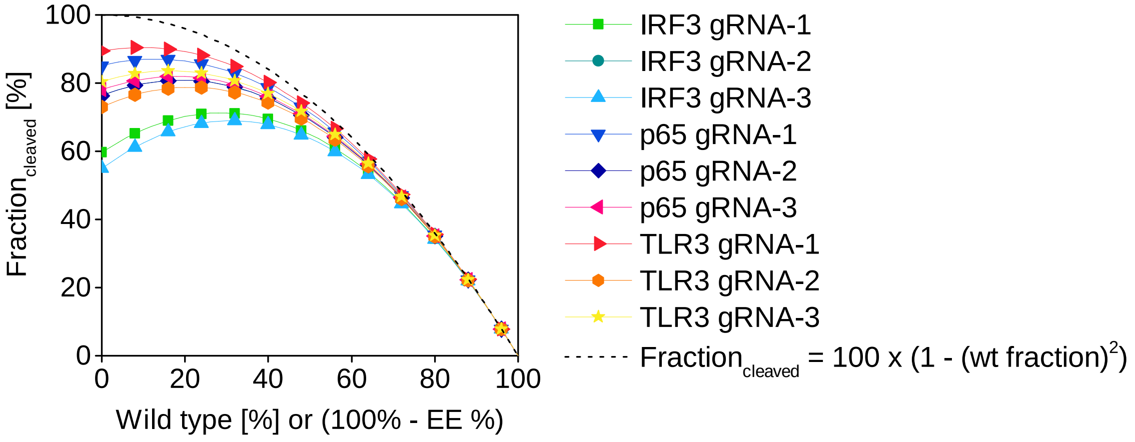

Figure S 9: Estimate of endonuclease mediated cleavage depending on the amount of wild type for experimentally observed indel patterns. To predict the cleavage for the different cell lines generated with the indicated gRNAs, we used the frequency of indels measured by the Sanger sequencing and the TIDE (Figure 3a main text). This allowed to calculate the probability to form homoduplexes for each indel. Note that for low amount of wild type sequence, cleavage would range between 60% and 90%. The dotted line represents the cleavage efficiency when assuming that wild type homoduplexes, but no mutant homoduplexes, can form.

Supplemental Figure 10

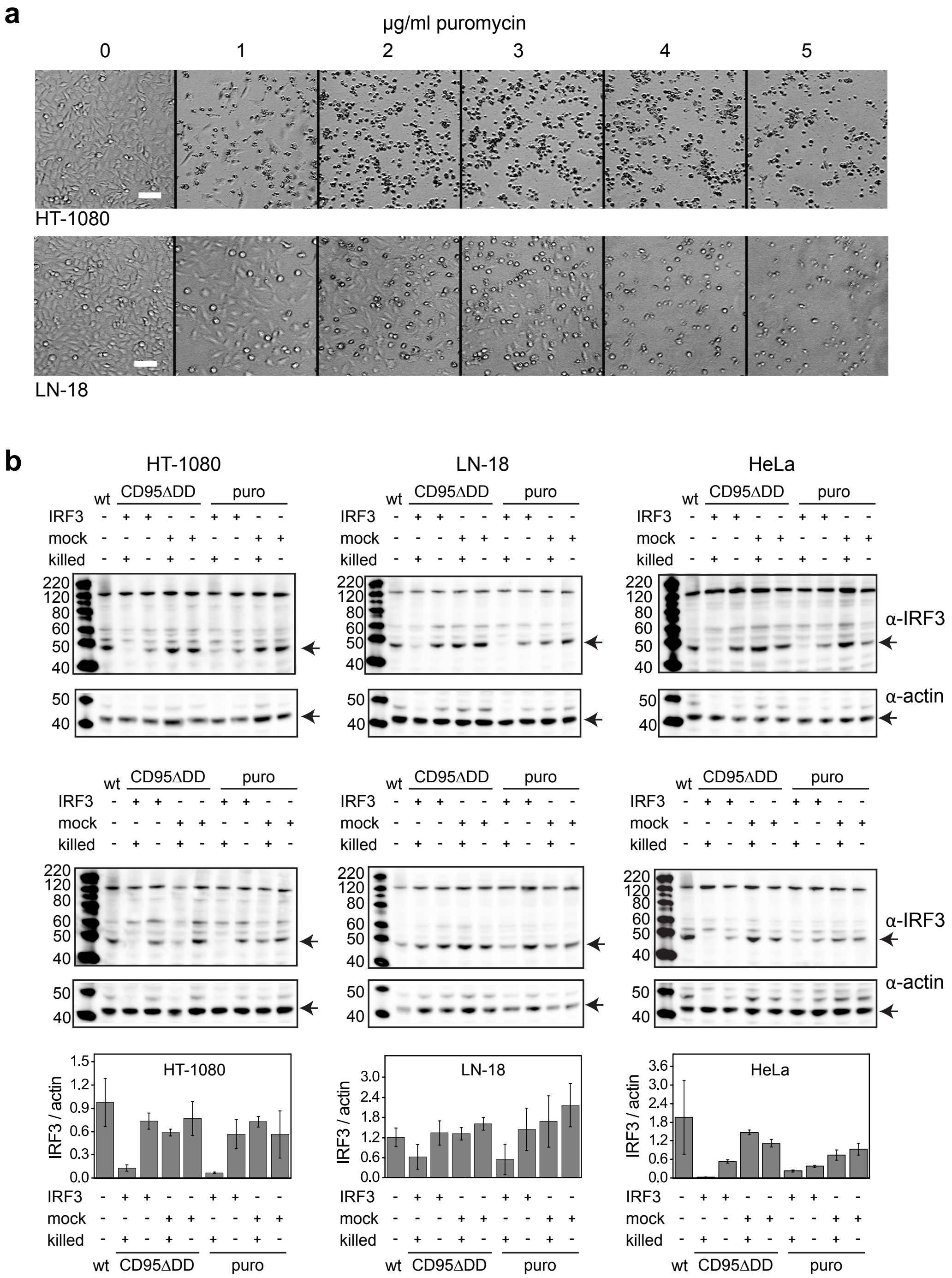

Figure S 10: Death receptor-based and puromycin-based enrichment of Cas-9 expressing cells. (**a**) Cell death in HT-1080 and LN-18 cells was assessed by microscopy 30 h upon continuous treatment with the indicated concentration of puromycin. 100% cell death after 30 h was seen for HT-1080 cells with 2 µg/ml and for LN-18 cells with 4 µg/ml. In the genome editing experiment, we then chose 3 and 5 µg/ml puromycin to efficiently kill non-transfected HT-1080 and LN-18 cells, respectively. Scale bar = 20 µm (**b**) Western blots of IRF3 protein. In two independent experiments, HT-1080, LN-18 and HeLa cells were transfected with the plasmids Cas9-2A-CD95ΔDD or Cas9-2A-puro coding also for gRNA-3 targeting the *IRF3* gene or gRNA targeting *GFP* (mock). To evaluate the enrichment effect, cells were treated with IZsCD95L and 5 µg/ml puromycin, respectively, or left untreated (+/- killed). To facilitate comparison, the mean and s.e.m of the ratio of IRF3 and actin protein signals from blots were plotted as bar diagram.

Supplemental Figure 11

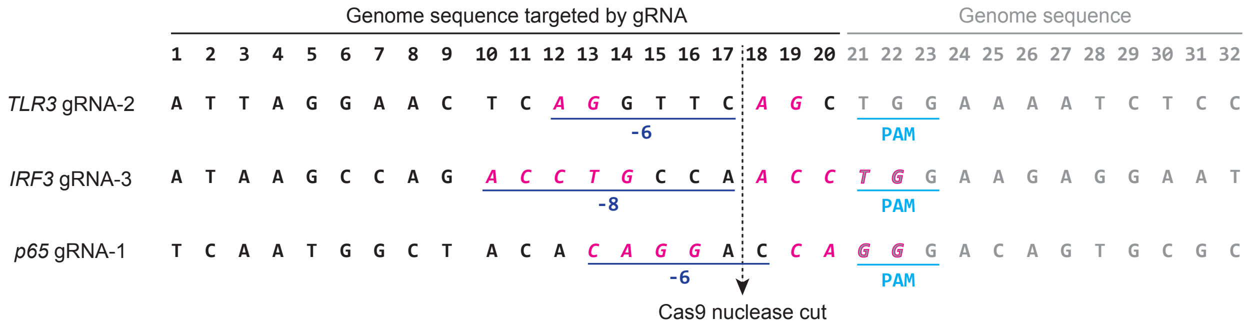

Figure S 11: The TIDE analysis provided information on the insertions and deletions that are introduced in a specific genomic region targeted by the gRNAs. Using *IRF3* gRNA-3, we observed the frequent and reproducible insertion of one nucleotide, the deletion of one nucleotide and the deletion of 8 nucleotides in three different cell lines (Figure 3 and 5d in the main text). This deletion of 8 nucleotides is compatible with the repeated presence of a 6 nucleotide sequence, separated by 8 nucleotides, on both sides of the cut. This microhomology domain sequence likely serves as a template for the repair. Two other examples of such repeated sequences are shown, a 2-nucleotide one for TLR3 gRNA-2 and a 4-nucleotide one for p65 gRNA-1, that are compatible with the observed indels of 6 nucleotides in both cases.

Supplemental Note - Editing Efficiency

The fraction of edited genes, denoted as the editing efficiency (EE), is typically derived from an endonuclease assay like the T7E1 or the Surveyor assay using the following formula:

$$EE = 1 - \sqrt{1 - fraction_{cleaved}} \qquad \text{(S-1)}$$

with $fraction_{cleaved}$ being the sum of the intensities of the two bands corresponding to the cleavage product of the endonuclease divided by the total intensities of bands of cleaved and non-cleaved products. This equation relies on several assumptions (see for example Guschin, 2010, Methods in molecular biology):

(i) Complete denaturation of the PCR products
(ii) Random re-annealing
(iii) A diversity of mutations that is so large that mutated strands cannot re-anneal with its perfectly complementary sequence.
(iv) Complete cleavage of the mismatches by the endonuclease

In this work, the sequencing of the PCR products clearly challenged the assumption (iii): the editing did not generate a random pattern with a very large diversity, but rather a small number of different indels with few indels representing the majority. Therefore, we took this into account to calculate the relationship between the genome editing and the fraction cleaved. For this, we introduced m different states corresponding to $m-1$ different indels generated by the specific gRNA and 1 wild-type state. Each state j can be found with a probability p_j, so that:

$$\sum_{j=1}^{m} p_j = 1$$

After denaturing n double stranded molecules of PCR product, we reanneal $2n$ single stranded ones, each of them with the probability p_j to be in each of the m states. The result of this reannealing can be either a homoduplex, which would appear in the non-cleaved product after the T7E1 assay, or a heteroduplex that would be cleaved by the endonuclease. The probability of forming a homoduplex is the probability of annealing two strands in the same state, so ${p_j}^2$ for each state j. The probability of forming any homoduplex is therefore the sum of all those probabilities:

$$P_{homoduplex} = \sum_{j=1}^{m} {p_j}^2 \qquad \text{(S-2)}$$

Hence, the probability of forming a heteroduplex, which is eventually measured as cleaved fraction, is:

$$fraction_{cleaved} \approx P_{heteroduplex} = 1 - P_{homoduplex} \qquad \text{(S-3)}$$

As shown in Supplemental Figures S9 and S10, a clear consequence of this is that even when the genome editing is complete, $P_{heteroduplex}$ is not equal to 1. Moreover, due to the parabolic and rather flat shape of the curves around their maximum, for low wild type percentages, so high editing efficiencies, the cleavage fraction is poorly sensitive to changes in efficiencies. On top, the amplitude of the expected cleavage fraction depends on the diversity of indels, which itself depends on the gRNA. Thus, for high efficiencies, the T7E1 assay can resonably predict that this efficiency is high, but cannot provide a good quantification for it.

When is equation (S-1) applicable? The assumption (iii) is a particular case where there is a large number m of possible indels, all with reasonably similar probabilities. A consequence of the large m, the probability for each of the $m-1$ indels will be small. The previous formula did not require the distinction between wild type and mutations, but let us now define $j = 1$ as the state of the wild-type. The probability of forming a hetero-duplex can be written with the contribution of wild type and mutations separately:

$$P_{heteroduplex} = 1 - {p_1}^2 - \sum_{j=2}^{m} {p_j}^2 \qquad \text{(S-4)}$$

If the fraction of wt is significantly larger than the fraction of mutant species and/or if m is very high with each p_j having similar values, then the last term can become negligible. Thus, the probability (or fraction) of wild type p_1, can be written as follows:

$$p_1 = \sqrt{1 - P_{heteroduplex}} \qquad \text{(S-5)}$$

With the editing efficiency being $1 - p_1$ and the measured cleavage fraction estimated with $P_{heteroduplex}$, the efficiency of editing can be written as in equation (S-1). From the simulation with different indel patterns (Supplemental Figures 9 and 10), we can estimate that equation (S-1) is valid for editing efficiencies below 30%. In this range, the T7E1 assay quantification has the advantage of amplifying the effect of the editing. Indeed, for low cleavage fraction, equation (S-1) becomes:

$$EE = 1 - \sqrt{1 - fraction_{cleaved}} \approx 1 - (1 - (\frac{1}{2} fraction_{cleaved})) = \frac{1}{2} fraction_{cleaved},$$

In other terms, in this case the cleavage fraction amplifies low editing efficiencies by a factor of 2.
